# The Effect of Mild Gestational Diabetes Mellitus Treatment on Adverse Pregnancy Outcomes: A Systemic Review and Meta-Analysis

**DOI:** 10.3389/fendo.2021.640004

**Published:** 2021-03-26

**Authors:** Samira Behboudi-Gandevani, Razieh Bidhendi-Yarandi, Mohammad Hossein Panahi, Mojtaba Vaismoradi

**Affiliations:** ^1^ Faculty of Nursing and Health Sciences, Nord University, Bodø, Norway; ^2^ Department of Biostatistics, University of Social Welfare and Rehabilitation Sciences, Tehran, Iran; ^3^ Department of Epidemiology, School of Public Health and Safety, Shahid Beheshti University of Medical Sciences, Tehran, Iran

**Keywords:** adverse pregnancy outcome, adverse maternal outcomes, adverse neonatal outcomes, mild gestational diabetes, treatment

## Abstract

**Objectives:**

It is uncertain whether the treatment of mild gestational diabetes mellitus (GDM) improves pregnancy outcomes. The aim of this systemic review and meta-analysis was to investigate the effect of mild GDM treatment on adverse pregnancy outcomes.

**Methods:**

A comprehensive literature search was conducted on the databases of PubMed, Scopus, and Google Scholar to retrieve studies that compared interventions for the treatment of mild GDM with usual antenatal care. The fixed/random effects models were used for the analysis of heterogeneous and non-heterogeneous results. Publication bias was assessed using the Harbord test. Also, the DerSimonian and Laird, and inverse variance methods were used to calculate the pooled odds ratio of events. The quality assessment of the included studies was performed using the Modified Newcastle–Ottawa Quality Assessment scale and the CONSORT checklist. In addition, the risk of bias was evaluated using the Cochrane Collaboration’s tool for assessing risk of bias.

**Results:**

The systematic review and meta-analysis involved ten studies consisting of 3317 pregnant women who received treatment for mild GDM and 4407 untreated counterparts. Accordingly, the treatment of mild GDM significantly reduced the risk of macrosomia (OR = 0.3; 95%CI = 0.3–0.4), large for gestational age (OR = 0.4; 95%CI = 0.3–0.5), shoulder dystocia (OR = 0.3; 95%CI = 0.2–0.6), caesarean-section (OR = 0.8; 95%CI = 0.7–0.9), preeclampsia (OR = 0.4; 95%CI = 0.3–0.6), elevated cord C-peptide (OR = 0.7; 95%CI = 0.6–0.9), and respiratory distress syndrome (OR = 0.7; 95%CI = 0.5–0.9) compared to untreated counterparts. Moreover, the risk of induced labor significantly increased in the treated group compared to the untreated group (OR = 1.3; 95%CI = 1.0–1.6). However, no statistically significant difference was observed between the groups in terms of small for gestational age, hypoglycemia, hyperbilirubinemia, birth trauma, admission to the neonatal intensive care unit, and preterm birth. Sensitivity analysis based on the exclusion of secondary analysis data was all highly consistent with the main data analysis.

**Conclusion:**

Treatment of mild GDM reduced the risk of selected important maternal outcomes including preeclampsia, macrosomia, large for gestational age, cesarean section, and shoulder dystocia without increasing the risk of small for gestational age. Nevertheless, the treatment could not reduce the risk of neonatal metabolic abnormalities or several complications in newborn.

## Introduction

Gestational diabetes mellitus (GDM) is defined as diabetes diagnosed in the second or third trimester of pregnancy without the presence of overt diabetes prior to gestation (1). GDM is one of the most common endocrinopathies among pregnant women and influences 4-12% of all pregnancies (2). Previous studies have demonstrated that GDM is associated with the increased risk of adverse feto-maternal and neonatal outcomes (3, 4). Therefore, its treatment can significantly reduce both the short- and long-term effects of GDM (5–9).

Lifestyle modifications including diet therapy, physical exercise, and pharmacological treatments such as oral antidiabetic medications and/or insulin are suggested for the treatment of GDM (1). However, there is an ongoing debate regarding the relationship between various perinatal risk factors and the milder form of GDM with glucose levels lower than the criteria for treatment. The Hyperglycemia and Adverse Pregnancy Outcomes’ (HAPO) study indicated that GDM is associated with the enhanced risk of adverse perinatal outcomes, without an obvious threshold at which the risk is increased (10). Therefore, the determination of an optimal threshold for the screening and treatment of mild GDM becomes difficult. Although it has been suggested that mild GDM is associated with the increased perinatal risk (11–13), it is uncertain whether the treatment of hyperglycemia exerts a protective influence on perinatal outcomes among pregnant women with GDM.

Studies on the treatment of mild GDM and its effect on adverse pregnancy outcomes have shown controversial results. A modest benefit from the identification and treatment of women with mild carbohydrate intolerance during pregnancy has been shown (12). On the other hand, a multicenter randomized trial by Landon et al. (12) on 958 pregnant women with mild GDM showed that the treatment of mild GDM did not significantly reduce the frequency of stillbirth or perinatal death, neonatal hypoglycemia, hyperbilirubinemia, birth trauma, and elevated cord-blood C-peptide level. However, it reduced the risk of macrosomia, shoulder dystocia, cesarean delivery, and pregnancy related hypertensive disorders (12). In contrast, pharmacological therapy neither reduced birth weight nor improved maternal or neonatal outcomes among women with mild GDM (14).

Given the lack of conclusive evidence regarding the benefits of the treatment of mild GDM, this systematic review and meta-analysis investigated the effect of mild GDM treatment on adverse pregnancy outcomes. Accordingly, the review question was as follows: does the treatment of mild GDM through diet therapy and antidiabetic medications improve adverse pregnancy outcomes in terms of maternal and neonatal adverse outcomes compared to untreated women?

## Material and Methods

This systematic review and meta-analysis was informed by the Preferred Reporting Items for Systematic Reviews and Meta-Analyses (PRISMA) (15). The review question was framed using the PICO statement as follows:

P: pregnant women with mild GDM; I: treatment through diet therapy and medications; C: treatment effect on adverse pregnancy outcomes; O: maternal and neonatal adverse outcomes.

The objectives of this review were to assess:

o the pooled risk of adverse single and composite maternal outcomes among those pregnant women who received the treatment of mild GDM compared to untreated counterparts;
o the pooled risk of adverse single and composite neonatal outcomes among those pregnant women who received the treatment of mild GDM compared to untreated counterparts;
o sensitivity analysis based on the exclusion of secondary analysis studies.

### Eligibility Criteria

Studies were identified eligible if: (I) they presented a clear definition of mild GDM or gestational hyperglycemia; (II) described the details of screening strategies and the threshold of blood sugar in screening tests; (III) reported at least one of the short-term single maternal and neonatal outcomes of GDM; (IV) reported the number or prevalence of adverse events; (V) described the treatment process; (VI) compared adverse pregnancy outcomes between those women who received treatment for mild GDM and untreated counterparts.

Exclusion criteria were non-original studies including reviews, commentaries, editorials, letters, meeting abstracts, case reports, and conference proceedings that did not provide accurate and clear data on research variables, and the presence of preexisting glucose intolerance or diabetes in studies’ samples.

### Search Strategy

A comprehensive literature search was conducted on the databases of PubMed (including Medline), Web of Science, and Scopus to retrieve original studies published in English language and on the prevalence and incidence of adverse maternal and neonatal outcomes among women with mild GDM up to May-2020. Further, a manual search in the references list of the selected studies and other relevant reviews was carried out to maximize the identification of eligible studies.

The following keywords, alone or in combination using the Boolean method, were used in the search: (adverse pregnancy outcomes OR pregnancy outcomes OR pregnancy complications OR gestational age OR macrosomia OR large for gestational age OR LGA OR small for gestational age OR SGA OR neonatal hypoglycemia OR hypoglycemia OR Hyperbilirubinemia OR icterus OR elevated C-peptide OR c-peptide OR C peptide OR NICU OR NICU admission OR respiratory distress syndrome OR RDS OR Apgar OR preterm birth OR preterm labor OR still birth OR IUFD OR intrauterine fetal death OR mortality OR IUGR OR intrauterine growth restriction OR polyhydramnios OR oligohydramnios OR preeclampsia OR pregnancy induced hypertension OR gestational hypertension OR PIH OR hemorrhage OR postpartum hemorrhage OR PPH OR placenta abruption OR decolman OR placenta previa OR antepartum hemorrhage OR maternal weight gain OR pregnancy weight gain OR gestational weight gain OR birth weight OR induction of labor OR labor induction OR induced labor OR instrumental delivery OR operative delivery OR cesarean sections OR C-section OR abdominal deliveries OR birth trauma OR shoulder dystocia) AND (mild gestational diabetes OR mild GDM OR mild gestational hyperglycemia OR mild maternal hyperglycemia OR mild glucose intolerance in pregnancy OR mild gestational glucose intolerance OR mild gestational carbohydrate intolerance OR mild carbohydrate intolerance in pregnancy OR mild gestational impaired glucose tolerance OR mild impaired glucose tolerance in pregnancy).

### Study Selection and Data Extraction

The screening of titles, abstracts and full texts of studies was conducted independently by two authors based on the eligibility criteria and the following data was extracted from eligible studies: the first author’s name; journal title; publication year; country; study design; sample size; population characteristics including age and body mass index (BMI); mild GDM screening strategy; mild GDM criteria and laboratory values of blood sugar tests; treatment details; quality assessment and outcome measurements including the number and prevalence of adverse outcomes. To prevent bias in the data extraction and data entry, the accuracy of data before the meta-analysis was assessed through double checking the data extraction process.

### Study Outcomes and Definition of Mild GDM

For the purpose of the present review, a composite outcome of adverse maternal outcomes and the important maternal events of labor induction, cesarean section, preeclampsia was selected. Also, a composite outcome of adverse neonatal outcomes and the single neonatal events of macrosomia, large for gestational age (LGA), small for gestational age (SGA), hypoglycemia, hyperbilirubinemia, elevated cord C-peptide, admission to the neonatal intensive care unit (NICU), respiratory distress syndrome (RDS), shoulder dystopia, and preterm birth was selected. Mild GDM was defined as any glucose intolerance during pregnancy, which was lower than the criteria for the diagnosis of GDM.

### Quality Appraisal and Risk of Bias

Quality of the selected studies was critically appraised in terms of the methodological structure and presentation of results. Two authors who were blinded to the study’s author and institution, and the journal’s title evaluated the quality of each study independently. The modified consolidated standards of reporting trials (CONSORT) as a validated quality assessment checklist for RCTs was used for the appraisal. Studies with scores ≥70% of the highest score of the CONSORT checklist were judged as high quality, 40–70% as moderate quality, 20–40% as low quality, and <20% as very low quality (16). The risk of bias in these studies was assessed using the Cochrane Collaboration’s tool for assessing risk of bias for RCTs (17). Accordingly, the risk of bias was categorized as ‘low risk’, ‘high risk’, and ‘unclear risk’.

### Statistical Analysis

The software package STATA (version 14; STATA Inc., College Station, TX, USA) was used for statistical data analysis. Heterogeneity between the studies was assessed using the I^2^ index and P < 0.05 was interpreted as heterogeneity. Heterogeneous and non-heterogeneous results were analyzed using the random/fixed effects models for calculating the pooled effect, respectively. Publication bias was assessed through the Harbord test. The DerSimonian and Laird, and inverse variance methods were used to calculate the pooled odds ratio (OR, 95% CI) of events. Meta-regression was performed to explore the effect of maternal age as the source of heterogeneity. In addition, sensitivity analysis was run to investigate the influence of each individual study on the overall meta-analysis summary estimate. A graph of the results of an influence analysis in which the meta-analysis was re-estimated omitting each study in turn was presented. P < 0.05 was set as significance level.

## Results

### Search Results, Study Selection, Study Characteristics, and Quality Assessment

**Figure 1:**
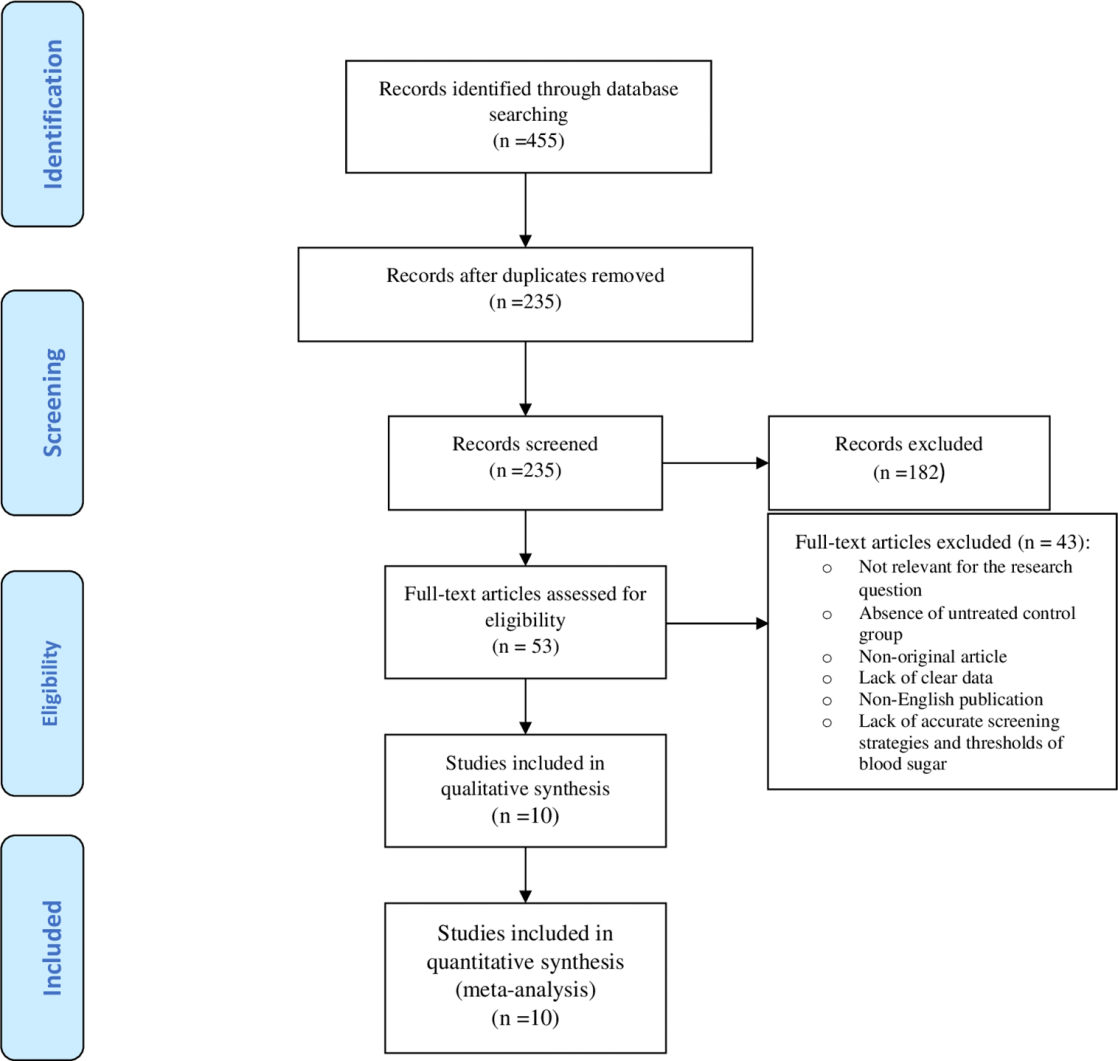
Flow diagram of the search strategy and study selection.

**Figure 1** illustrates the flow diagram of the search strategy and study selection. The search strategy yielded 455 potentially relevant articles, but 53 articles were identified for further full-text assessment based on the inclusion criteria. Finally, 10 studies were selected to conduct the meta-analysis consisting of 3317 pregnant women who received treatment for mild GDM, and 4407 untreated counterparts. **Table 1** shows a summary of the studies assessing the risk and prevalence of adverse perinatal outcomes in the groups.

**Table 1:** The characteristic of the studies selected for the systematic review and meta-analysis.

Author, year	Country	Screening test	Mild GDM diagnostic criteria	Mild GDM treated group characteristics	Mild GDM untreated group characteristics	Type of treatment
Bahado-Singh et al. (18)	USA	GCT-50 g-1h followed by OGTT-100 g-3h	FBS< 95 mg/dL and two or more values of BS-1h >180 mg/dL, BS-2h >155 mg/dL, BS-3h >140 mg/dL	*1.* with male fetus: n= 244, Age: -, BMI: - *2.* with female fetus: n= 233, Age: -, BMI: -	*1.* with male fetus: n= 225, Age: -, BMI: - *2.* with female fetus: n= 230, Age: -, BMI: -	Diet therapy and insulin, if needed
Berggren et al. (19)	USA	GCT-50 g-1h followed by OGTT-100 g-3h	*1.* GCT 50 g-1h, 130-200 mg/dL *2.* FBS< 95 mg/dL and two or more values of BS-1h >180 mg/dL, BS-2h >155 mg/dL, BS-3h >140 mg/dL	*2.* Hispanic: n= 274, Age: 29.5 (5.7), BMI: 30.3 (4.4) 2. Non-Hispanic: n= 123, Age: 29.2 (5.9), BMI: 29.7 (5.5)	*1.* Hispanic: n= 520, Age: 27.6 (5.4), BMI: 30.1 (4.5) *1.* Non-Hispanic: n= 247, Age: 28 (65.4), BMI: 29.5 (5.3) *2.* Hispanic: n= 225, Age: 29.5 (5.6), BMI: 30.2 (4.3) *2.* Non-Hispanic: n= 116, Age: 28.5 (5.0), BMI: 30.6 (6.2)	Diet therapy and insulin, if needed
Blackwell et al. (20)	USA	GCT-50 g -1h followed by OGTT-100 g-3h	FBS< 95 mg/dL and two or more values of BS-1h >180 mg/dL, BS-2h >155 mg/dL, BS-3h >140 mg/dL	*1.* Normal Weight Gain: n= 257, Age: 29.5 (5.7), BMI: 25.3 (22.1–28.7) 2. Excessive Weight *Gain:* n= 174, Age: 28.8 (5.8), BMI: 26.5 (23.7–29.4)	*1.* Normal Weight Gain: n= 188, Age: 29.8 (5.5), BMI: 24.7 (22.3–28.8) *2.* Excessive Weight Gain: n= 222, Age: 28.5 (5.4), BMI: 26.0 (23.4–28.7)	Diet therapy and insulin, if needed
Bo et al. (21)	Italy	GCT-50 g-1h followed by OGTT-100 g-3h	*1.* Only one abnormal value in OGTT 100g: FPG >95 mg/dL or BS-1h > 180 mg/dL or BS-2h > 155 mg/dL or BS-3h > 140 mg/dL *2.* GCT ≥140 mg/dL and OGTT 100g-negative	*1.* n= 100, Age: 32.9 (4.7), BMI: 25.6 (5.4)	2. n= 350, Age: 31.8 (4.3), BMI: 23.5 (4.8)	Diet therapy and insulin, if needed
Bonomo et al. (22)	Italy	GCT-50 g-1h followed by OGTT-100 g-3h	GCT ≥140 mg/dL and OGTT 100g- negative	n= 150, Age: 31.1 (4.7), BMI: 23.1 (4.4)	n= 150, Age: 30.7 (5.1), BMI: 23.0 (4.5)	Diet therapy
Casey et al. (14)	USA	GCT-50 g-1h followed by OGTT-100 g-3h	FBS < 105 mg/dL and at least two elevated values on BS-1h > 190 mg/dL, BS-2h > 165 mg/dL, BS-3h > 145 mg/dL	n= 189, Age: 31.3 (6), BMI: 29.0 (4.8)	n= 186, Age: 31.2 (6), BMI: 28.9 (5.3)	Diet therapy and glyburide
Landon et al. (23)	USA	GCT-50 g-1h followed by OGTT-100 g-3h	FBS< 95 mg/dL and two or more values of BS-1h >180 mg/dL, BS-2h >155 mg/dL, BS-3h >140 mg/dL	n= 264, Age: 29.2 (5.2), BMI:-	n= 236, Age: 28.7 (5.5), BMI:-	Diet therapy and insulin, if needed
Landon et al. (12)	USA	GCT-50 g -1h followed by OGTT-100 g-3h	FBS< 95 mg/dL and two or more values of BS-1h >180 mg/dL, BS-2h >155 mg/dL, BS-3h >140 mg/dL	n= 485, Age: 29.2 (5.7), BMI:-	n= 473, Age: 28.9 (5.6), BMI:-	Diet therapy and insulin, if needed
Moss et al. (24)	Australia	OGTT-75 g- 2h	FBS < 140 mg/dL and BS-2h: 140-198 mg/dL	n= 474, Age:-, BMI:-	n= 496, Age:-, BMI:-	Diet therapy and insulin, if needed
Sugiyama et al. (25)	Japan	GCT-50 g -1h followed by OGTT-75 g-2h	One elevated value for FBS ≥100 mg/dL, BS-1h ≥ 180 mg/dL, BS-2h ≥ 150 mg/dL	*1.* n= 172, Age: 34.5 (4.8), BMI: 22.6 (5.3) *2.* n= 178, Age: 33.7 (4.7), BMI: 22.7 (4.8)	n= 543, Age: 33.7 (4.9), BMI: 21.6 (6.1)	Diet therapy and insulin, if needed

BMI, body mass index; GCT, glucose challenge test; OGTT, oral glucose challenge test; FBS, fasting blood sugar; BS, blood sugar.

The quality assessment of the included studies has been presented in **Supplementary Table 1**. Five studies were classified as high quality (12, 18–20, 23), 3 as moderate quality (14, 21, 25), 2 as low quality (22, 24), but no study had very low quality. All studies (12, 14, 18–25) had the interventional design, of which 3 (33.3%) studies were secondary analysis (18–20). Also, 6 studies were conducted in the USA (12, 14, 18–20, 23), two in Italy (21, 22), one in Japan (25) and one in Australia (24). All of studies used glucose tolerance test (GCT)-50 g-1h followed by oral glucose tolerance test (OGTT)-100 g-3h as the screening and diagnostic test (12, 14, 18–23, 25), except one that used OGTT-75 g-2h (24).

### Meta-Analysis of Outcomes

**Table 2** shows the pooled OR of adverse maternal and neonatal outcomes, heterogeneity and publication bias estimation in those women who received the treatment of mild GDM compared to untreated women.

**Table 2:** Heterogeneity and publication bias estimation and meta-analysis for the risk of adverse maternal and neonatal outcomes among the treated women with mild gestational diabetes compared to the untreated women.

Outcome	Sample size		Publication bias, Harbord test	Heterogeneity(I^2^%)	Pooled OR (95% CI)*
	Mild Untreated GDM	Mild Treated GDM			
**Composite adverse maternal outcomes**	10768	8765	0.065	62.7	**0.8 (0.7-0.9)**
Cesarean section	3497	2829	0.586	0.0	**0.8 (0.7, 0.9)**
Labor induction	2678	1966	0.652	58.7	**1.3 (1.0, 1.6)**
Shoulder dystocia	2678	1966	0.881	0.0	**0.3 (0.2, 0.6)**
Preeclampsia	1729	1815	0.293	0.0	**0.4 (0.3, 0.6)**
**Composite adverse neonatal outcomes**	31510	23322	0.624	62	**0.7 (0.6-0.8)**
Macrosomia	4299	3265	0.882	0.0	**0.3 (0.3, 0.4)**
LGA	4873	3609	0.869	43.3	**0.4 (0.3, 0.5)**
SGA	3788	3259	0.216	0.0	1.1 (0.9, 1.4)
Hypoglycemia	3274	2191	0.484	0.0	1.0 (0.8, 1.2)
Hyperbilirubinemia	3746	2429	0.273	16.8	0.9 (0.7, 1.1)
Elevated cord c-peptide	2180	1850	0.138	32.4	**0.7 (0.6, 0.9)**
Birth trauma	1096	1141	0.497	0.0	0.5 (0.2, 1.1)
NICU admission	3966	2857	0.273	45.0	0.9 (0.7, 1.2)
RDS	2345	1404	0.348	0.0	**0.7 (0.5, 0.9)**
Preterm birth	1943	1317	0.749	73.4	0.9 (0.8, 2.4)

GDM, Gestational Diabetes; LGA, Large for Gestational Age; SGA, Small for Gestational Age; NICU, neonatal intensive care unit; RDS, Respiratory Distress Syndrome.

*Bold values indicate statistical significance.

**Figure 2:**
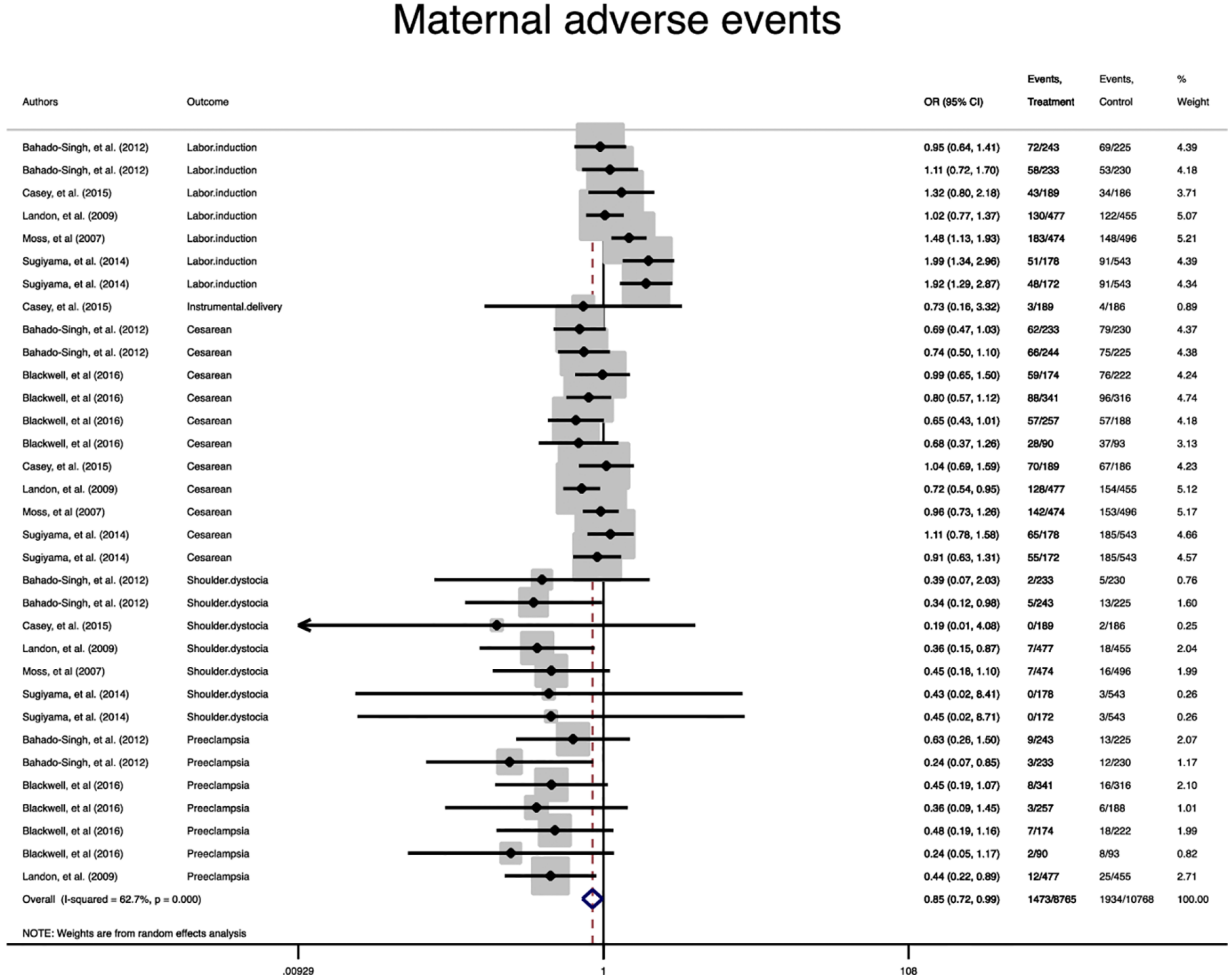
Forest plot of pooled odds ratio of composite adverse maternal outcome.

A statistically significant difference between the treatment group and the control group in the risk of the composite maternal and neonatal perinatal outcomes was observed (Pooled OR = 0.8, 95% CI = 0.7-0.9) and (Pooled OR = 0.7, 95% CI = 0.6-0.8), respectively, (**Figures 2** **,** **3**).

**Figure 3:**
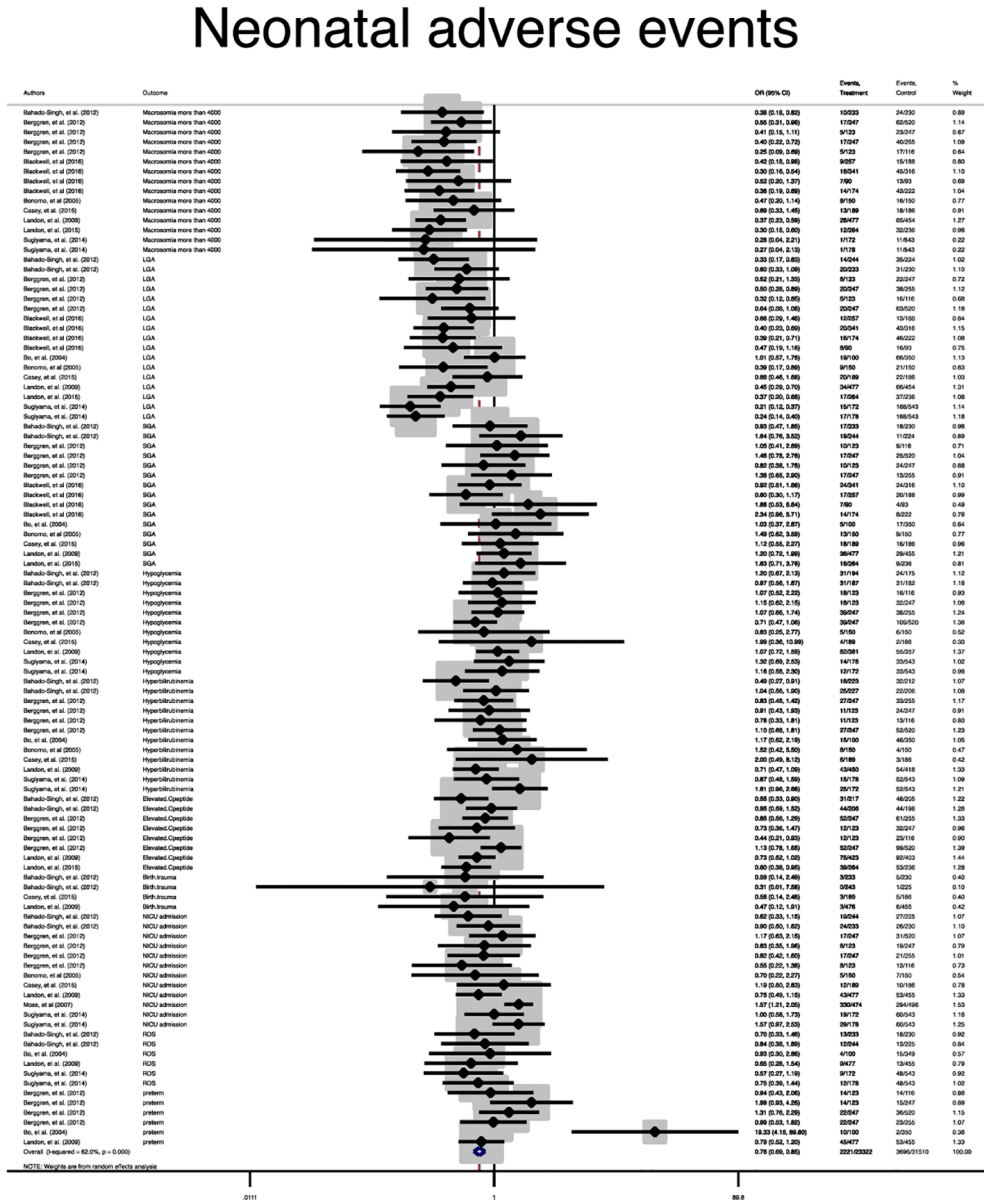
Forest plot of pooled odds ratio of composite adverse neonatal outcome.

In term of maternal outcomes, the pooled OR of cesarean section (Pooled OR = 0.8, 95% CI = 0.7-0.9) and shoulder dystocia (Pooled OR = 0.3, 95% CI = 0.2-0.6) as well as preeclampsia (Pooled OR = 0.4, 95% CI = 0.3-0.6) were significantly reduced in the treated group compared with the untreated group. In contrast, the pooled OR of labor induction among the treated women was significantly 1.3 folds higher than that of the untreated women (Pooled OR = 1.3, 95% CI = 1.0-1.6) (**Table 2**, **Supplementary Figures 1**–**4**).

**Figure 4:**
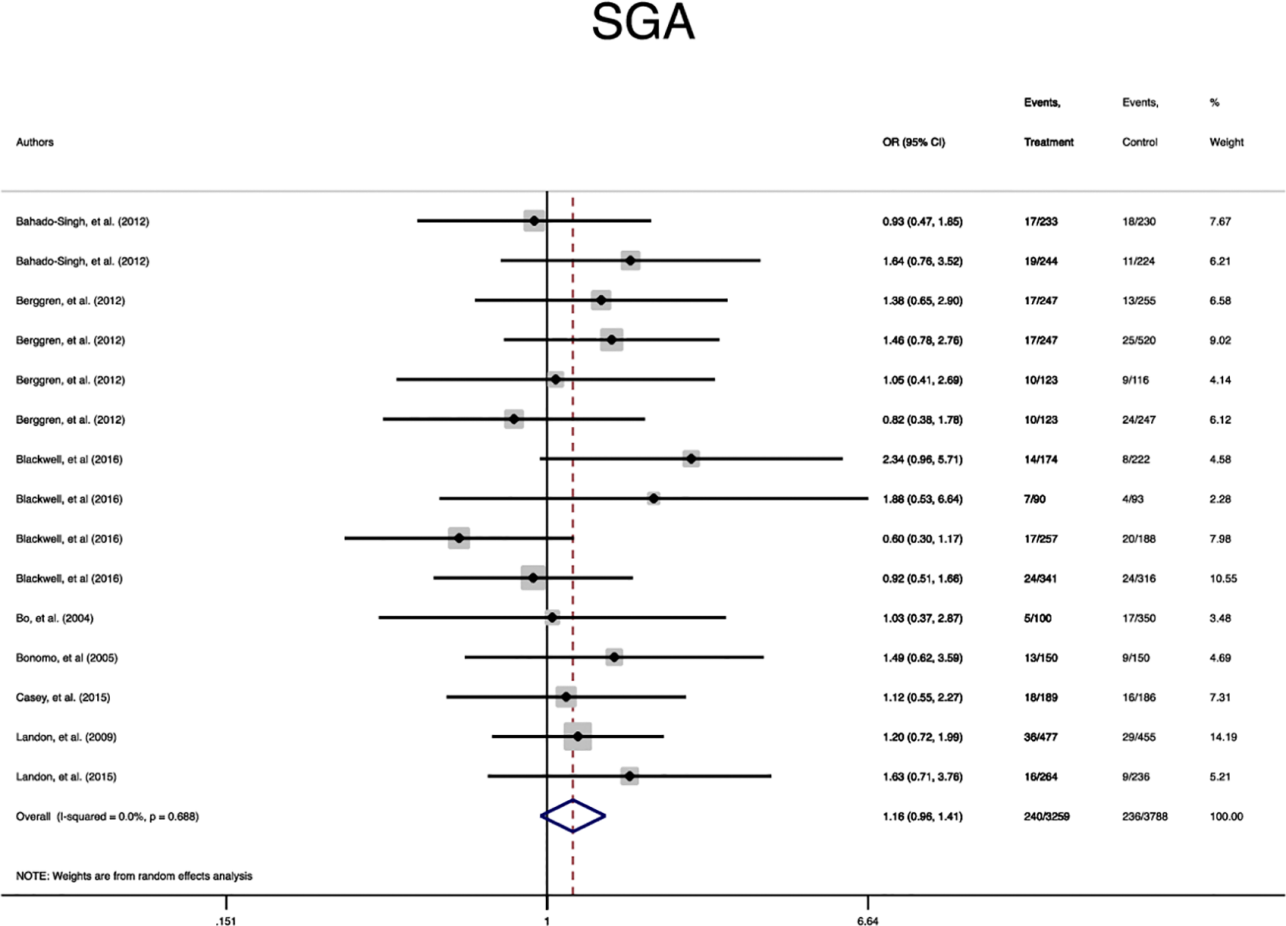
Forest plot of pooled odds ratio of small for gestational age.

In terms of neonatal outcomes, the adverse events of SGA, hypoglycemia, hyperbilirubinemia, birth trauma, admission to the NICU, and preterm birth were not significantly different between the groups (**Table 2**, **Supplementary Figures 5**–**9**). However, the risk of macrosomia (Pooled OR = 0.3, 95% CI = 0.3-0.4), LGA (Pooled OR = 0.4, 95% CI = 0.3-0.5), elevated cord C-Peptide (Pooled OR = 0.7, 95% CI = 0.6-0.9) and RDS (Pooled OR = 0.7, 95% CI = 0.5-0.9) among the treated women were significantly lower than that of the untreated women (**Table 2**, **Figures 4** **–** **6**, **Supplementary Figures 5**–**11**). According to meta-regression, the reported ORs were not influenced by maternal age (**Supplementary Figure 12**).

**Figure 5:**
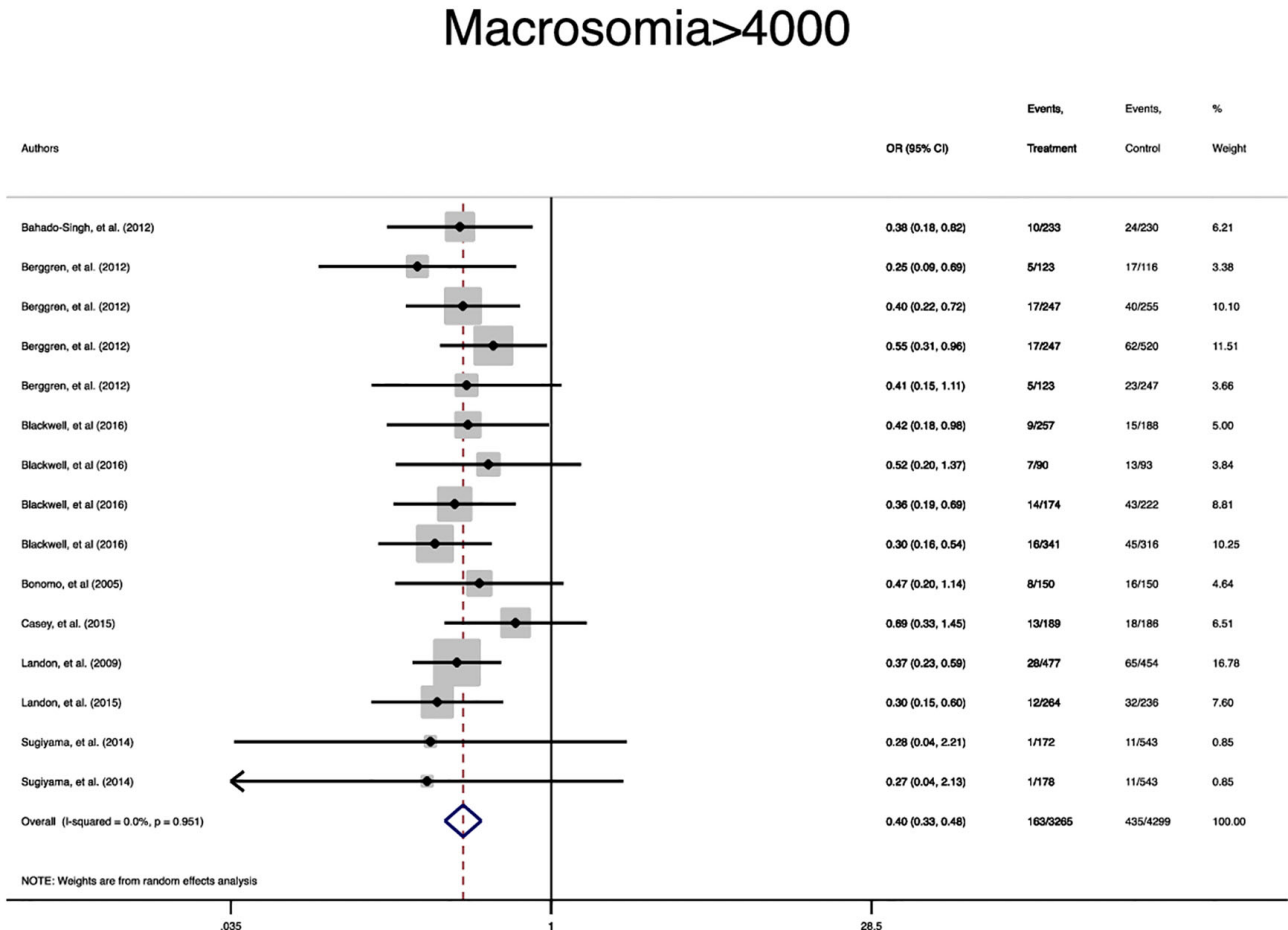
Forest plot of pooled odds ratio of macrosomia.

**Figure 6:**
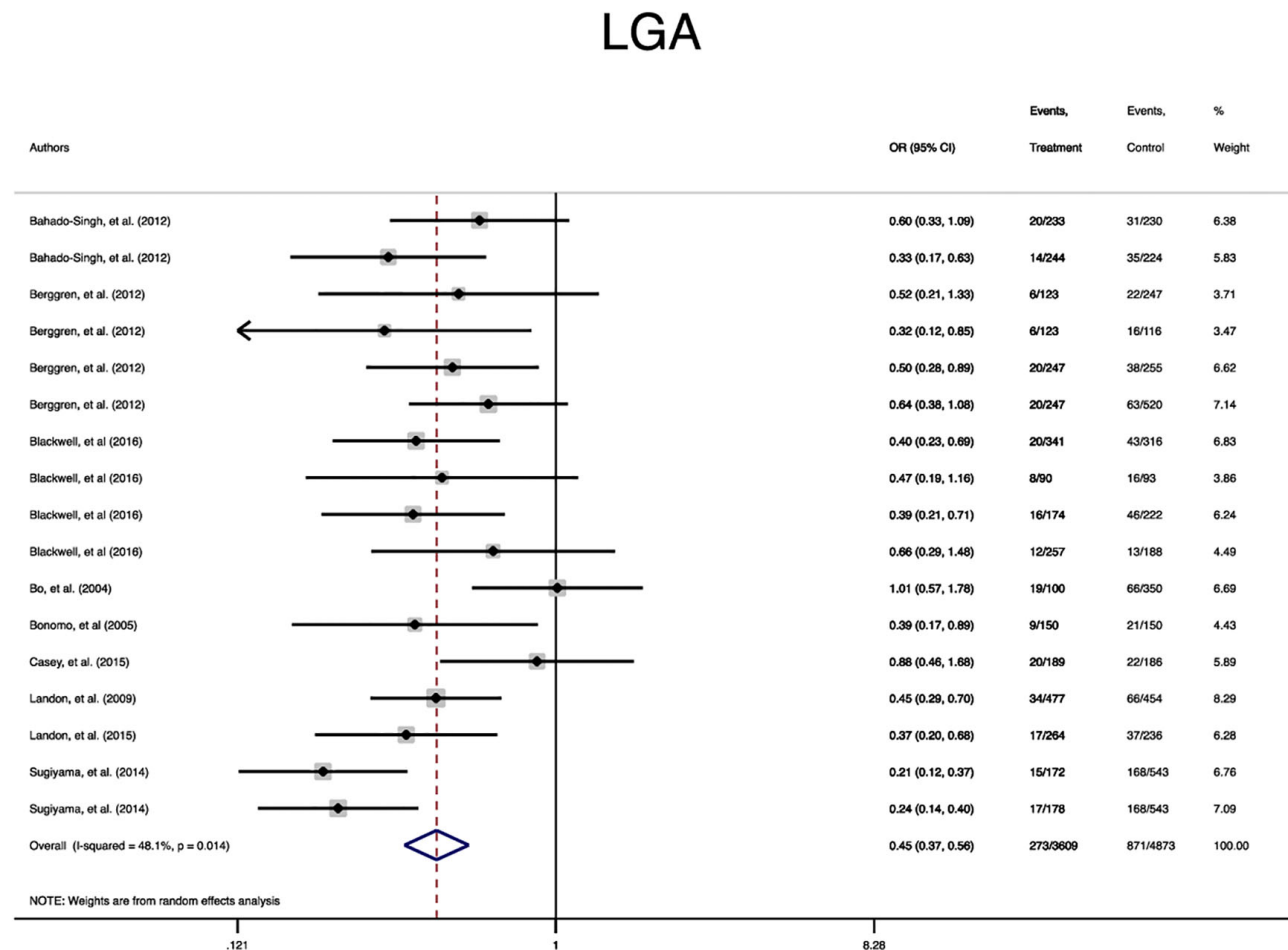
Forest plot of pooled odds ratio of large for gestational age (LGA).

### Sensitivity Analysis

Since three of the included studies used a secondary analysis design, sensitivity analysis was performed after their exclusion from the data analysis process. The results were highly consistent with the main data analysis’ results (**Supplementary Figures 13 A–Q**).

### Publication Bias and Risk of Bias

No substantial publication bias for meta-analyses based on the Harbord test was observed (**Tables 2**). The included studies mostly were judged as having a low risk of bias for the evaluated domains (**Supplementary Figure 13**). Accordingly, all studies had a low risk of bias in the reporting of selective outcomes and incomplete outcome data, but about 60% of them had an unclear risk of bias and 20% had a high risk of bias in the blinding of personnel, participants, and outcome assessment. Also, 10% had a high risk or unclear risk of bias in random sequence generation or allocation.

## Discussion

The results of the current systematic review and meta-analysis showed that although the treatment of mild GDM did not reduce the risk of selected neonatal outcomes including hypoglycemia, hyperbilirubinemia, birth trauma, admission to the NICU, and preterm birth, it reduced the risks of macrosomia, LGA, elevated cord C-Peptide and RDS as well as maternal outcomes of cesarean section, shoulder dystocia and preeclampsia, without causing any significant increase in the risk of SGA. However, the risk of labor induction among the treated women was higher than the untreated women.

Despite the unquestionable evidence and the wide range of recommendations regarding the benefit of treatment for women with GDM (6, 26, 27), there is an ongoing debate about the effect of the treatment for glucose intolerance and a lower than diagnostic thresholds of GDM. Notably, after the publication of the results of the HAPO’s study demonstrating that maternal glycemia continuously was associated with the increasing risk of adverse perinatal outcomes, with no obvious threshold at which risks increase, the debate as to whether a benefit exists for the treatment of the milder form of GDM assumes even greater importance now than in the past.

Diet therapy along with glucose monitoring was mentioned as the primary therapeutic choice in all studies meeting our inclusion criteria for inclusion to our meta-analysis. Pharmacotherapy and mainly insulin therapy were initiated if dietary modification failed to control glycemic levels. The treatment significant decreased the risk of adverse outcomes related to fetal overgrowth including macrosomia and LGA, which in turn reduced the risk of shoulder dystocia and caesarean section among the treated women with mild GDM compared to untreated counterparts. It is suggested that maternal hyperglycemia therapy can regulate fetal hyperinsulinemia and subsequently decrease the body fat storage in fetus (28). It should be noted that although the treatment increased the risk of SGA as a sign of overtreatment, the increased risk had no statistically significant difference between the treated women and the untreated women. Meanwhile, the risk of induced labor showed a significant increase in the treatment group compared to the untreated group. It is hypothesized that knowledge about treatment allocation may have influenced the decisions of healthcare providers involved in the study (29). As well, the treatment of mild GDM decreased the risk of preeclampsia, as a major complication of pregnancy, in the treated women compared to the untreated women. This may be due to improvements in vascular changes such as arteriosclerosis and glomerular filtration dysfunction caused by altered carbohydrate metabolism in the background of insulin resistance (30). Moreover, our review showed the modest benefit of treatment in the risk reduction of RDS among the newborns of the treated women compared to the untreated women. It might be attributed to the decreased risk of cesarean section and preterm birth in the mild GDM group, but preterm birth showed a non-significant reduction in the treatment group compared to the untreated group.

Our review findings showed that treatment significantly decreased the elevated cord C-peptide as a marker of fetal hyperinsulinemia (31), but it did not decrease the risk of metabolic outcomes in newborns. Similarly, we noticed that GDM treatment had no significant impact on the risk of other adverse neonatal outcomes such as SGA, admission to the NICU and preterm birth. It can be attributed in part to the fact that those outcomes are most consistently associated with more severe glucose intolerance than those that have been present in the participants included to our study. In this respect, most participants with mild GDM in our meta-analysis had normal fasting maternal glucose, but a threshold for an increased risk of clinical neonatal hypoglycemia might not be apparent until fasting maternal glucose levels exceeded 100 mg/dL (5.6 mmol/L) (10, 12).

This is the largest and the most updated systematic review and meta-analysis of RCTs conducted on the most stringent method of determining whether a cause-effect relationship exists between the intervention and outcome. In addition, the included studies showed consistency across all outcomes of interest and no statistically significant publication bias was identified. However, some limitations should be considered during the interpretation of the results. For instance, given a lack of a uniform standard for defining mild GDM during pregnancy, individual studies on the topic has produced a varying definition for mild GDM, implying that women with different degrees of glucose intolerance are involved. A lack of unique definition for each adverse pregnancy outcome might have affected the review findings. Most of the included studies had a small sample size and the results were dominated by the secondary analysis of one study (12). However, for further confirmation and avoidance of any bias, the sensitivity analysis was performed through excluding the secondary data. All included studies were conducted in developed counties and the results might not be extrapolated to developing countries that might have differences in the lifestyle and healthcare standards. Also, the ethnicity as the source of heterogenicity could not be assessed given the lack of related information in the studies. Moreover, subgroup-analysis based on the results of fasting maternal glucose, as an important indicator of adverse pregnant outcomes, could not be performed (10), because defined mild GDM criteria in the studies mostly had normal fasting blood sugar. Since most of the studies used diet therapy and/or insulin therapy, the effect of various treatments of mild GDM on adverse pregnancy outcomes between the groups could not be compared. We could not achieve enough power to report significant results for some outcomes including perinatal/neonatal mortality and intrauterine fetal death.

In conclusion, the treatment of mild GDM using diet therapy and insulin is likely to reduce the risk of several key adverse outcomes of interest such as macrosomia and LGA, shoulder dystocia, cesarean section, preeclampsia as well as newborn RDS, without causing any significant increase in the risk of SGA. Also, our review findings indicated that the treatment of mild GDM might not reduce other adverse neonatal outcomes. Further studies with larger sample sizes are required in different population and ethnicities to examine the effect of treatment on other important adverse pregnancy outcomes with low prevalence.

## Data Availability Statement

The original contributions presented in the study are included in the article/**Supplementary Material**. Further inquiries can be directed to the corresponding author.

## Author Contributions

SB-G conceptualized the study and was involved in study design, search in databases, study selection, data extraction, drafting the manuscript, and revising it critically for important intellectual content. MV was involved in manuscript drafting, editing, and revising it critically for important intellectual content. RB-Y contributed to quality assessment, data analysis, and interpreting data. MP contributed to quality assessment, data analysis, and interpreting data. All authors contributed to the article and approved the submitted version.

## Funding

Nord University, Bodø, Norway covered the article processing charge.

## Conflict of Interest

The authors declare that the research was conducted in the absence of any commercial or financial relationships that could be construed as a potential conflict of interest.
