# Supplementary material for: The Effect of Mild Gestational Diabetes Mellitus Treatment on Adverse Pregnancy Outcomes: A Systemic Review and Meta-Analysis
Source: Front Endocrinol (Lausanne). 2021 Mar 26;12:640004. doi: 10.3389/fendo.2021.640004 (PMC8033156; doi:10.3389/fendo.2021.640004)
Supplement: Supplementary file 1 [file DataSheet_1.docx]

Supplementary Table 1. Quality assessment of the included studies using the Consort Assessment Scale for interventional studies

| Author | Methods | | | | | | | | | | | | | | | | |
| --- | --- | --- | --- | --- | --- | --- | --- | --- | --- | --- | --- | --- | --- | --- | --- | --- | --- |
|  | Trial design | | Participants | | Interventions | Outcomes | | Sample size | | Randomization | | Allocation concealment mechanism | Implementation | Blinding | | Statistical methods | |
|  | a | b | a | b |  | a | b | a | b | a | b |  |  | a | b | a | b |
| Bahado-Singh, et al. (2012) | + | + | + | + | + | + | + | + | + | + | + | + | + | - | - | + | + |
| Berggren, et al. (2012) | + | + | + | + | + | + | + | + | + | + | + | + | + | - | - | + | + |
| Blackwell, et al. (2016) | + | + | + | + | + | + | + | + | + | + | + | + | + | - | - | + | + |
| Bo, et al. (2004) | - | - | + | + | + | + | - | - | - | - | - | - | - | + | + | + | + |
| Bonomo, et al. (2005) | - | - | + | + | + | + | - | - | - | - | - | - | - | - | - | + | + |
| Casey, et al. (2015) | - | - | + | + | + | + | - | - | - | - | - | - | - | + | + | + | - |
| Landon, et al. (2015) | + | + | + | + | + | + | + | + | + | + | + | + | + | - | - | + | + |
| Landon, et al. (2009) | + | + | + | + | + | + | + | + | + | + | + | + | + | - | - | + | + |
| Moss, et al. (2007) | - | - | + | + | + | + | - | + | - | - | - | - | - | - | - | + | - |
| Sugiyama, et al. (2014) | - | - | + | + | + | + | + | - | - | - | - | - | - | - | - | + | + |

| Author | Results | | | | | | | | | | Total | Quality |
| --- | --- | --- | --- | --- | --- | --- | --- | --- | --- | --- | --- | --- |
|  | Participant flow (a diagram is strongly recommended) | | Recruitment | | Baseline data | Numbers analyzed | Outcomes and estimation | | Ancillary analyses | Harms |  |  |
|  | a | b | a | b |  |  | a | b |  |  |  |  |
| Bahado-Singh, et al. (2012) | + | + | + | + | + | + | + | + | + | - | 24 | High |
| Berggren, et al. (2012) | + | + | + | + | + | + | + | + | + | - | 24 | High |
| Blackwell, et al. (2016) | + | + | + | + | + | + | + | + | + | - | 24 | High |
| Bo, et al. (2004) | - | - | - | - | + | + | + | + | + | + | 14 | Moderate |
| Bonomo, et al. (2005) | - | - | - | - | + | + | + | + | - | - | 10 | Low |
| Casey, et al. (2015) | + | - | - | - | + | + | + | + | - | - | 12 | Moderate |
| Landon, et al. (2015) | + | + | + | + | + | + | + | + | + | - | 24 | High |
| Landon, et al. (2009) | + | + | + | + | + | + | + | + | + | - | 24 | High |
| Moss, et al. (2007) | - | - | - | - | + | + | + | + | - | - | 10 | Low |
| Sugiyama, et al. (2014) | + | + | + | + | + | + | + | + | + | + | 18 | Moderate |

Supplementary figure 1. Forest plot of pooled odds ratio of cesarean section.

Supplementary figure 2. Forest plot of pooled odds ratio of shoulder dystocia.

Supplementary figure 3. Forest plot of pooled odds ratio of preeclampsia.

Supplementary figure 4. Forest plot of pooled odds ratio of labor induction.

Supplementary figure 5. Forest plot of pooled odds ratio of hypoglycemia.

Supplementary figure 6. Forest plot of pooled odds ratio of hyperbilirubinemia.

Supplementary figure 7. Forest plot of pooled odds ratio of birth trauma.

Supplementary figure 8. Forest plot of pooled odds ratio of NICU admission.

Supplementary figure 9. Forest plot of pooled odds ratio of preterm birth.

Supplementary figure 10. Forest plot of pooled odds ratio of elevated c-peptide.

Supplementary figure 11. Forest plot of pooled odds ratio of RDS.

.

Supplementary figure 12. Bubble plot of the meta-regression relationships between adverse pregnancy outcomes and maternal age.


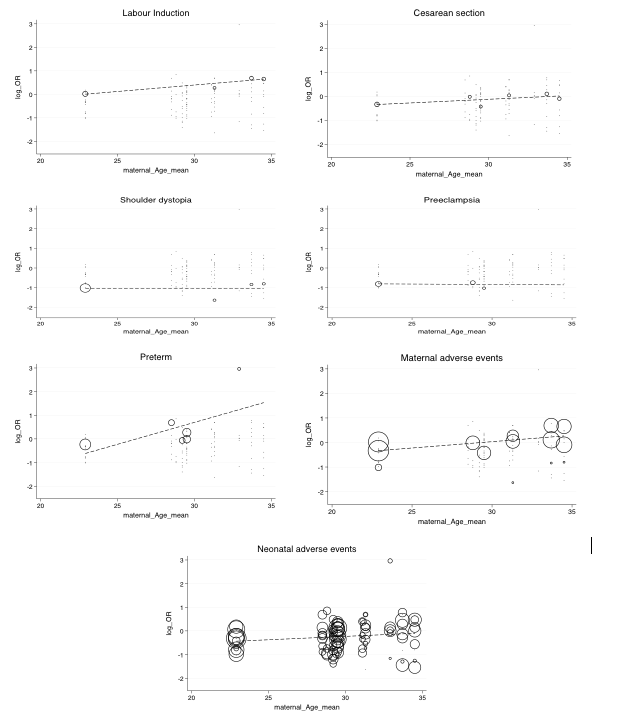


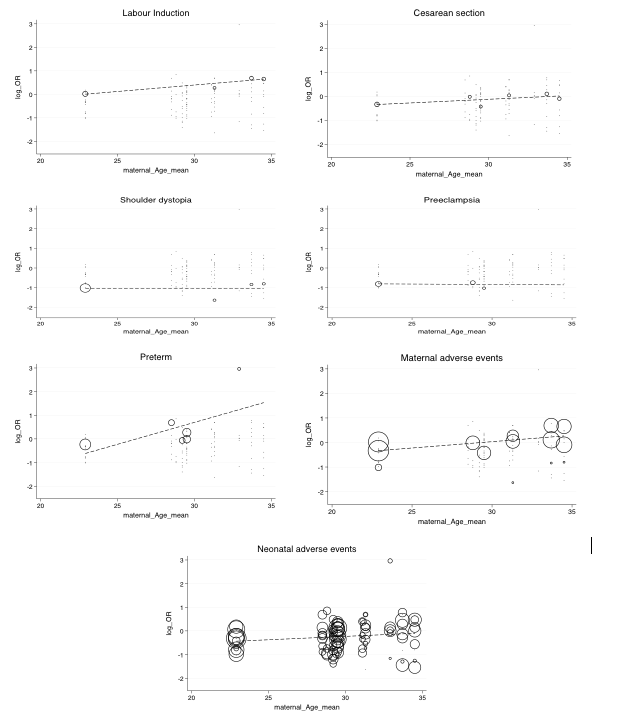


Supplementary figure 13. Sensitivity analysis (A-Q)

**A:** Sensitivity analysis for **macrosomia**

**B:** Sensitivity analysis for **LGA**

**C:** Sensitivity analysis for **SGA**

**D:** Sensitivity analysis for **elevated c-peptide**

**E:** Sensitivity analysis for **hypoglycemia**

**F:** Sensitivity analysis for **hyperbilirubinemia**

**G:** Sensitivity analysis for **birth trauma**

**H:** Sensitivity analysis for **NICU**

**I:** Sensitivity analysis for **RDS**

**J:** Sensitivity analysis for **labor induction**

**K:** Sensitivity analysis for **cesarean section**

**L:** Sensitivity analysis for **shoulder dystopia**

**M:** Sensitivity analysis for **preeclampsia**

**O:** Sensitivity analysis for **preterm**

**P:** Sensitivity analysis for **neonatal adverse events**


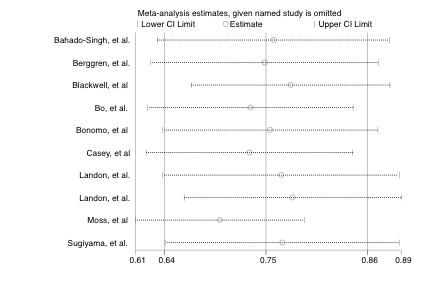


**Q:** Sensitivity analysis for **maternal adverse events**

Supplementary figure 13. Risk of bias in interventional studies.

A:

| Author, year | Bias in random sequence generation | Bias in allocation concealment | Bias in blinding of participants and personnel | Bias in blinding of outcome assessment | Bias in incomplete outcome data | Bias in selective outcome  reporting |
| --- | --- | --- | --- | --- | --- | --- |
| Bahado-Singh, et al. (2012) | 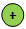 | 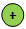 | 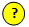 | 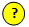 | 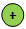 | 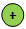 |
| Berggren, et al. (2012) | 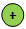 | 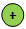 | 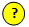 | 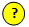 | 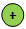 | 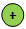 |
| Blackwell, et al. (2016) | 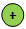 | 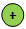 | 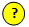 | 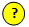 | 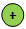 | 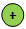 |
| Bo, et al. (2004) | 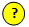 | 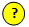 | 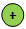 | 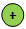 | 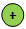 | 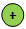 |
| Bonomo, et al. (2005) | 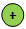 | 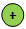 | 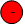 | 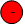 | 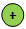 | 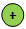 |
| Casey, et al. (2015) | 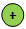 | 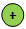 | 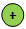 | 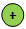 | 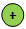 | 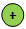 |
| Landon, et al. (2015) | 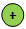 | 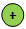 | 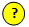 | 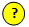 | 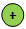 | 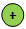 |
| Landon, et al. (2009) | 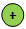 | 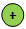 | 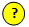 | 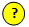 | 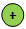 | 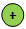 |
| Moss, et al. (2007) | 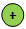 | 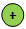 | 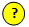 | 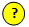 | 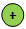 | 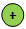 |
| Sugiyama, et al. (2014) | 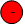 | 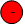 | 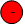 | 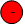 | 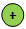 | 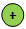 |
| 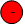 Yes (high risk of bias) 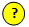 Unclear 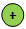 No (low risk of bias)  **RCT** | | | | | | |

B:
